# Gs- versus Golf-dependent functional selectivity mediated by the dopamine D_1_ receptor

**DOI:** 10.1038/s41467-017-02606-w

**Published:** 2018-02-05

**Authors:** Hideaki Yano, Ning-Sheng Cai, Min Xu, Ravi Kumar Verma, William Rea, Alexander F. Hoffman, Lei Shi, Jonathan A. Javitch, Antonello Bonci, Sergi Ferré

**Affiliations:** 1 National Institute on Drug Abuse, National Institutes of Health, Baltimore, MD 21224 USA; 2 Department of Psychiatry, College of Physicians & Surgeons, Columbia University, New York, NY 10032 USA; 3 Division of Molecular Therapeutics, New York State Psychiatric Institute, New York, NY 10032 USA

## Abstract

The two highly homologous subtypes of stimulatory G proteins Gαs (Gs) and Gαolf (Golf) display contrasting expression patterns in the brain. Golf is predominant in the striatum, while Gs is predominant in the cortex. Yet, little is known about their functional distinctions. The dopamine D_1_ receptor (D1R) couples to Gs/olf and is highly expressed in cortical and striatal areas, making it an important therapeutic target for neuropsychiatric disorders. Using novel drug screening methods that allow analysis of specific G-protein subtype coupling, we found that, relative to dopamine, dihydrexidine and N-propyl-apomorphine behave as full D1R agonists when coupled to Gs, but as partial D1R agonists when coupled to Golf. The Gs/Golf-dependent biased agonism by dihydrexidine was consistently observed at the levels of cellular signaling, neuronal function, and behavior. Our findings of Gs/Golf-dependent functional selectivity in D1R ligands open a new avenue for the treatment of cortex-specific or striatum-specific neuropsychiatric dysfunction.

## Abstract (web summary)

D1-like dopamine receptors are coupled to Golf proteins in the dorsal striatum but Gs in cortical and other areas. Here, the authors demonstrate selective agonism of Gs-coupled versus Golf-coupled D1 receptors.

## Introduction

Functional selectivity is defined as the ability of a ligand to demonstrate a biased profile of potency or efficacy on different signaling pathways. This is distinguished from prototypical uniform activation by general agonism^1,2^ produced by endogenous ligands. In recent years, many ligands with functionally selective properties for G-protein-coupled receptors (GPCRs) have emerged based on the concept that ligands can stabilize specific receptor conformations to which different signaling proteins, such as G proteins and β-arrestins, couple^1^. In addition, accessory proteins to the receptor^3^ as well as effector proteins^4^ may exert bias in signaling events exhibiting many potential sites for functional selectivity. Thus the concept of functional selectivity has provided a new avenue for the development of drugs with safer therapeutic index, when therapeutic and unwanted side effects are dependent on different signaling pathways^5^.

Several examples of functional selectivity have been reported for dopamine receptor ligands. Dopamine receptors are classified into Gs/Golf-coupled D_1_-like receptors (D1R and D5R) and Gi/o-coupled D_2_-like receptors (D2R, D3R and D4R). With respect to D_2_-like receptors, both G-protein-biased^6,7^ and β-arrestin-biased^8,9^ agonists have been characterized. With respect to D_1_-like receptors, biased agonism at G-protein versus β-arrestin signaling has also been reported^10–12^. We recently found differences in dopamine potency in promoting the coupling of different Gαi and Gαo (Gi/o) protein subtypes to the D2R, D3R, and D4R^13^. These results suggest the possibility of selectively targeting D_2_-like receptor in different brain areas relying on the predominant local expression of certain Gi/o proteins. However, there is no compelling evidence for a differential distribution of Gi/o proteins in the brain. This is in contrast with the clearly distinct distribution of the two subtypes of stimulatory G proteins, Gs and Golf. Golf is by far the most expressed and functions as a signaling G-protein for D1R in the striatum^14^, while Gs is predominantly expressed in cortical and other areas^15,16^.

In the present study, using a series of novel pharmacological assays, we addressed the possibility of Gs/olf protein subtype-dependent biased agonism of D1R ligands. Dihydrexidine (DHX) and N-propyl-apomorphine (NPA) behaved as full D1R agonists when coupled to Gs and as partial D1R agonists when coupled to Golf. The significant efficacy bias for Gs-mediated versus Golf-mediated signaling of DHX was further demonstrated with cellular signaling, electrophysiological and psychomotor activation experiments, which enhances our understanding of Golf-signaling in striatal function and psychomotor activity. Moreover, our results highlight the potential use of such functionally selective agonists for treating the “negative” cognitive symptoms of schizophrenia^17^.

## Results

### Gs- and Golf-biased engagement and activation by D1R ligands

**Fig. 1:**
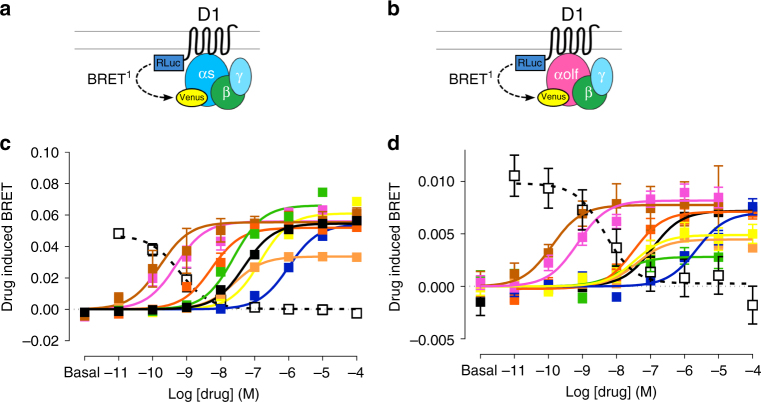
**a** Scheme for the engagement BRET between D1R-Rluc and Gs-Venus. **b** Scheme for the engagement BRET between D1R-Rluc and Golf-Venus. **c** Dose-response curves of drug-induced BRET between D1R-Rluc and Gs-Venus (black dopamine, blue norepinephrine, light orange SKF38393, dark orange SKF81297, brown SKF82958, yellow NPA, green DHX, magenta A77636, black open SCH23390 + 10^−6^ SKF81297). **d** Dose-response curves of BRET between D1R-Rluc and Golf-Venus (same color scheme). The error bars represent S.E.M

Using the receptor-Gα subunit engagement BRET configuration, the potencies and efficacies of different classes of D1R agonists were compared to dopamine for Gs and Golf coupling (Fig. 1 and Table 1). While the majority of the compounds behaved similarly for the engagement of Gs and Golf, two compounds, dihydrexidine (DHX) and N-propyl apomorphine (NPA), behaved quite differently (Fig. 1c, d; green and yellow curves, respectively). Notably, whereas these compounds behaved as full agonists (relative to DA) for Gs coupling (*E*_max_; DHX, 121.3%; NPA, 111.9%), they displayed only partial agonism for Golf coupling (*E*_max_; DHX, 39.0%; NPA, 67.9%; Fig. 1 and Table 1). To further validate the partial efficacy of DHX for Golf engagement, DHX was tested for its ability to counteract the agonist effect of dopamine (1 µM; Supplementary Fig. 1B). Although much less potent than the commonly used D1R antagonist SCH23390, the *I*_max_ value of DHX was in agreement with its *E*_max_ value (Supplementary Fig. 1B). The relative potency and efficacy of the different agonists were further tested with the Gα-γ activation BRET configuration (Supplementary Table 1). Similar to results obtained with the engagement assay, DHX and NPA exhibited significantly lower *E*_max_ values relative to DA for Golf activation (46.8 and 52.6%; Supplementary Table 1) while retaining *E*_max_ values comparable to DA for Gs activation.

**Table 1:** Pharmacological comparison of Gs and Golf engagement in D1R

	D1L_GsV		D1L_GolfV	
	EC_50_ (nM)	*E*_max_ (%)	EC_50_ (nM)	*E*_max_ (%)
DA	49.0 ± 4.3	100.0 ± 1.3	111.9 ± 25.4	100.0 ± 3.9
NE	1002.3 ± 116.3^c^	99.2 ± 2.2	2280.3 ± 784.5^c^	97.8 ± 7.9
SKF38393	25.1 ± 4.0	61.6 ± 1.3^c^	40.6 ± 14.6	62.1 ± 4.2^c^
SKF81297	5.4 ± 1.2^c^	95.0 ± 2.7	32.6 ± 12.7	98.8 ± 7.4
SKF82958	0.2 ± 0.1^c^	101.5 ± 5.2	0.1 ± 0.1^c^	107.6 ± 11.0
NPA	145.5 ± 19.5^b^	111.9 ± 2.4^b^	34.7 ± 17.3	67.9 ± 6.7^b^
DHX	24.3 ± 3.5	121.3 ± 2.7^c^	13.4 ± 8.0^a^	39.0 ± 6.1^c^
A77636	0.5 ± 0.1^c^	102.5 ± 3.8	0.7 ± 0.2^c^	113.4 ± 5.6

Data were fit by non-linear regression to a sigmoidal dose-response relationship against the agonist concentration. EC_50_ and *E*_max_ values are means ± S.E.M. of more than five experiments performed in triplicate. *E*_max_ values are expressed in % normalized to dopamine results

^a^ *p* < 0.05

^b^ *p* < 0.01

^c^ *p* < 0.001 compared with DA (one-way analysis of variance, followed by post hoc Tukey test)

Although β-arrestin is essential for GPCR internalization and desensitization, it can also transduce MAPK activation for various receptors^18^, including D1R^19^. Using the same luciferase-fused D1R construct used for the Gα engagement BRET assays, we measured agonist-induced recruitment of Venus-fused β-arrestin-2 to the D1R (Supplementary Table 1). GPCR kinase 2 (GRK2) was co-transfected to enhance β-arrestin-2 recruitment. SKF81297 and SKF38393 showed reduced and minimal β-arrestin-2 recruitment, respectively, confirming their recently reported biased G-protein- versus β-arrestin-dependent signaling^12^, while A77636 and SKF82958 maintained their high potencies and efficacies in the β-arrestin-2 recruitment assay. DHX and NPA behaved similarly to Gs engagement in terms of efficacy and potency and DHX also showed a significantly higher *E*_max_ value than DA (142.4%; Supplementary Table 1). Altogether, these results clearly indicate that DHX and NPA possess biased agonism toward Gs versus Golf but not toward Gs protein versus β-arrestin selectivity.

### Gα -D1R interface contribution to bias between Gs and Golf

**Fig. 2:**
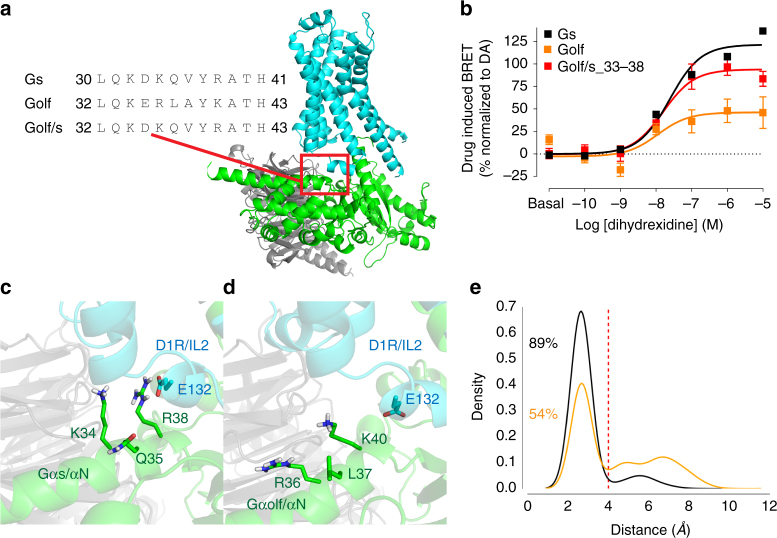
**a** Homology modeling of Gs (green) coupling to D1R (cyan) based on β2AR-Gs crystal structure (PDB: 3SN6) with the amino acid alignment among Gs (top row), Golf (middle row), and Golf/s_33–38 chimera (bottom row). **b** Dose-response curves of DHX-induced BRET between D1R-Rluc and Gs-Venus, Golf-Venus, or Golf/s_33–38-Venus (black, orange, and red respectively). BRET values are normalized to *E*_max_ values obtained by DA with corresponding Gα-Venus constructs. **c**, **d** Close-up views of the simulated interface between D1R and Gs (**c**) or D1R and Golf (**d**) at the intracellular loop 2 (IL2) of D1R and N-terminal α-helix (αN) of Gα subunit. **e** Frequency density distribution plot of 50 ns simulation for D1R-Gs (black) and D1R-Golf (orange). Proportions of salt bridge occurance (<4 Å) in the simulation are shown in percentage. The error bars represent S.E.M.

We then inspected the sequence differences between Gs and Golf to identify a potential structural element that is responsible for the observed biased agonism. Most of the motifs that interact with D1R are nearly identical between Gs and Golf. However, five residues are divergent at the end of αN helix of G-protein (residues 33–38 in Gs) apposing to the intracellular loop 2 (IL2) of D1R (Fig. 2). The impact of this divergence on the protein-protein interaction was then investigated both in vitro and in silico. To understand the functional impact of this divergence in the D1R-G-protein coupling, a Golf/s chimeric construct was made whereby an ERLAYK to DKQVYR mutation was introduced to the Golf-Venus construct (Fig. 2a). The effect of DHX in the D1R-Gα engagement was then tested with this construct. DHX-induced BRET changes were normalized to the *E*_max_ values obtained by DA with the corresponding Gα-Venus constructs (Fig. 2b). Similar to the results described above, Gs and Golf coupling were 123.3% and 46.8% *E*_max_ relative to DA, confirming the biased selectivity of this ligand. In contrast, DHX coupling to the Golf/s chimera was significantly increased (*E*_max_ = 94.0% relative to DA). A partial but significant increase in *E*_max_ was also observed with NPA (Supplementary Fig. 1D). Thus, our results suggest that the Gα/αN-D1R/IL2 interface is responsible for the biased agonism of DHX on D1R-Gs vs D1R-Golf. As expected, a full agonist effect was observed in Golf/s chimera coupling nearly the same as Gs or Golf with SKF 81297 (Supplementary Fig. 1C). Further, to exclude the possibility of effects from other non-cognate coupling, Gi1 and Gq engagement to the D1R was tested (Supplementary Fig. 1E, F). DA, SKF81297, or DHX did not cause any coupling of Gi1 or Gq while D2R-Gi1 coupling by DA and M1R-Gq coupling by carbachol were observed as positive controls (Supplementary Fig. 1E, F).

Next, the underlying molecular mechanism for the bias was explored with comparative homology modeling and molecular dynamics simulation. In the simulation of the D1R-Gs and D1R-Golf homology models constructed from β2AR-Gs complex (see Methods), we found that in the D1R-Gs complex, R38 of Gs forms a steady salt bridge interaction with E132 of D1R/IL2 (Fig. 2c), for which the simulation shows the interaction (<4 Å) holds for 89% of the time (Fig. 2e). In comparison, the D1R-Golf complex has a weaker salt bridge interaction between K40 of Golf and E132 of D1R/IL2 (Fig. 2d) with an intermittent interaction (<4 Å) of 54% within the simulated time (Fig. 2d). This reduced stability in salt bridge interaction between Golf/αN and D1R/IL2 is consistent with the lower signal observed in D1R-Golf BRET assay.

### Gs-biased agonism of DHX at the cellular signaling level

**Fig. 3:**
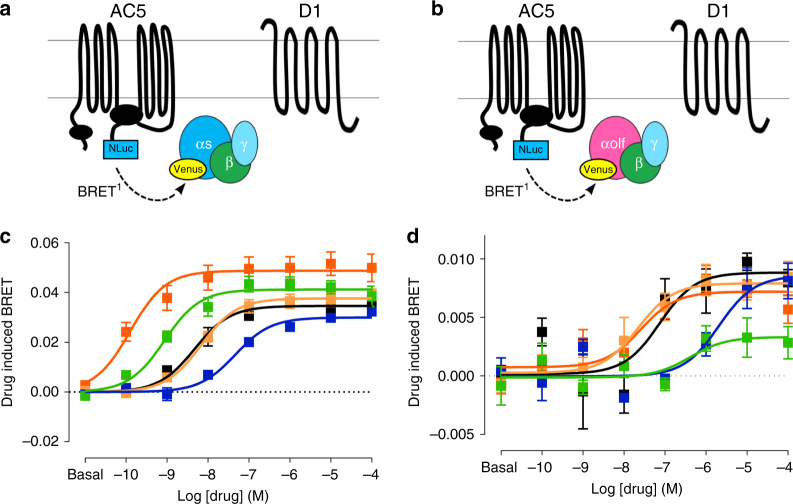
**a**. Scheme for the drug-induced interaction BRET between AC5-Nluc and Gs-Venus. **b** Scheme for the drug-induced interaction BRET between AC5-Nluc and Golf-Venus. **c** Dose-response curves of drug-induced BRET between AC5-Nluc and Gs-Venus (black DA, blue norepinephrine, light orange SKF38393, dark orange SKF81297, green DHX, black open SCH23390 + 10^−^^6^ SKF81297). **d** Dose-response curves of drug-induced BRET between AC5-Nluc and Golf-Venus (same color scheme). The error bars represent S.E.M.

To confirm the partial agonism of Golf-dependent signaling, DHX was tested in a novel Gα-AC5 coupling assay (Fig. 3 and Supplementary Table 2) in which D1R agonist-induced relative movement (BRET changes) between AC5 and Gs or Golf can be monitored. Assay optimization was first performed by testing various Gs and Golf biosensor constructs with different insertion positions (Supplementary Fig. 2). Ligand-induced BRET changes indicate relative conformational changes between Gs or Golf and AC5, reflecting AC5 activation level. D1R ligands were analyzed for the interactions between Gs and Golf and AC5 (Fig. 3). Again, DHX behaved as a more efficacious agonist than DA with Gs (*E*_max_ 119.0%), and as a partial agonist with Golf (*E*_max_ 37.6%; Suppl. Table 2 and Fig. 3). To confirm the results and validate this novel assay, we analyzed adenylate cyclase enzymatic activity, cAMP accumulation, in a unique lymphoma cell line (S49 cyc- cells) lacking Gs (Supplementary Fig. 3)^20^. In this cell line Gs- or Golf-dependent cAMP activation can be separately analyzed by rescuing the expression of either Gα protein subunit. In D1R electroporated cells, a selective full agonist SKF81297 and a selective partial agonist SKF38393 were used as reference compounds to compare with DHX. As expected, when compared to SKF81297 and SKF38393, *E*_max_ value for DHX yielded a similar partial efficacy value as SKF38393 with Golf co-transfection (45.1 and 50.3%, respectively), while maintaining partial and near full efficacy values with Gs co-transfection (67.4 and 87.6%, respectively). Forskolin was added to confirm G-protein-independent activation of endogenous AC (Supplementary Fig. 3C, mosaic bar). Increased cAMP production was observed in Golf or Gs electroporated cells compared to the mock electroporated cells due to AC5 co-electroporation in Golf and Gs cells (Supplementary Fig. 3C, solid and hatch bars vs. mosaic bar). In non-electroporated S49 cyc- cells, DHX or isoproterenol (β-adrenergic receptor agonist) was added to demonstrate the lack of Gs/olf proteins, as there was no AC activation (Supplementary Fig. 3D, green mosaic and purple mosaic). The effect of isoproterenol was rescued by Golf or Gs electroporation (Supplementary Fig. 3D, purple solid and hatch bars). Finally, a BRET-based cAMP biosensor was used to verify the specificity and potency of SKF81297 and DHX in HEK293T cells (Supplementary Fig. 4A, B, E, F). Propranolol (1 µM) was added to inhibit DA activation of β-adrenergic receptors (Supplementary Fig. 4E, F, black curves). The results demonstrate the lack of agonist activity for SKF81297 and DHX in non-D1R transfected cells (Supplementary Fig. 4A, E) and high potency and efficacy of SKF81297 and DHX in D1R-transfected cells (Supplementary Fig. 4B, F).

### Gs-biased agonism of DHX in mouse brain tissue

Previous reports have shown contrasting patterns of Gs and Golf expression in the brain, with Golf enriched in the striatum but not in the cortex and vice versa for Gs^15^. Indeed, using single cell RT-PCR analysis in Drd1-tdTomato BAC reporter mice, we were able to confirm a preferential expression of Gs and Golf genes in the prelimbic region of medial prefrontal cortex (mPFC) and the shell of the nucleus accumbens (NAc), respectively (Supplementary Fig. 5A, B). Virtually the same results were obtained with tissue punches, confirming the same differential gene expression when also including non-D1R-expressing cells (Supplementary Fig. 5C, D). Electrophysiological studies in mouse slices from Gs-enriched mPFC and Golf-enriched NAc were performed to confirm the predicted low efficacy of DHX in the striatum. The D1R agonist SKF81297 was used for comparison and expected to behave with full efficacy in both preparations. Both compounds have been reported to bind to D_2_-like receptors albeit with lower affinity than D1R^21,22^. Since D_2_-like receptors, especially D3R, co-localize with D1R in the brain^23^, the ability of both ligands to activate D2R and D3R was also evaluated in BRET-based functional assays. DHX and SKF81297 were about one order of magnitude less potent and less efficacious at D2R and D3R-mediated Go activation than at D1R-mediated Gs activation (Supplementary Figs. 4C, D, G, H, 6). Nevertheless, the non-selective D_2_-like receptor antagonist eticlopride was co-applied with DHX or SKF81297 to completely isolate D1R agonist-mediated effects in the brain slice preparation.

**Fig. 4:**
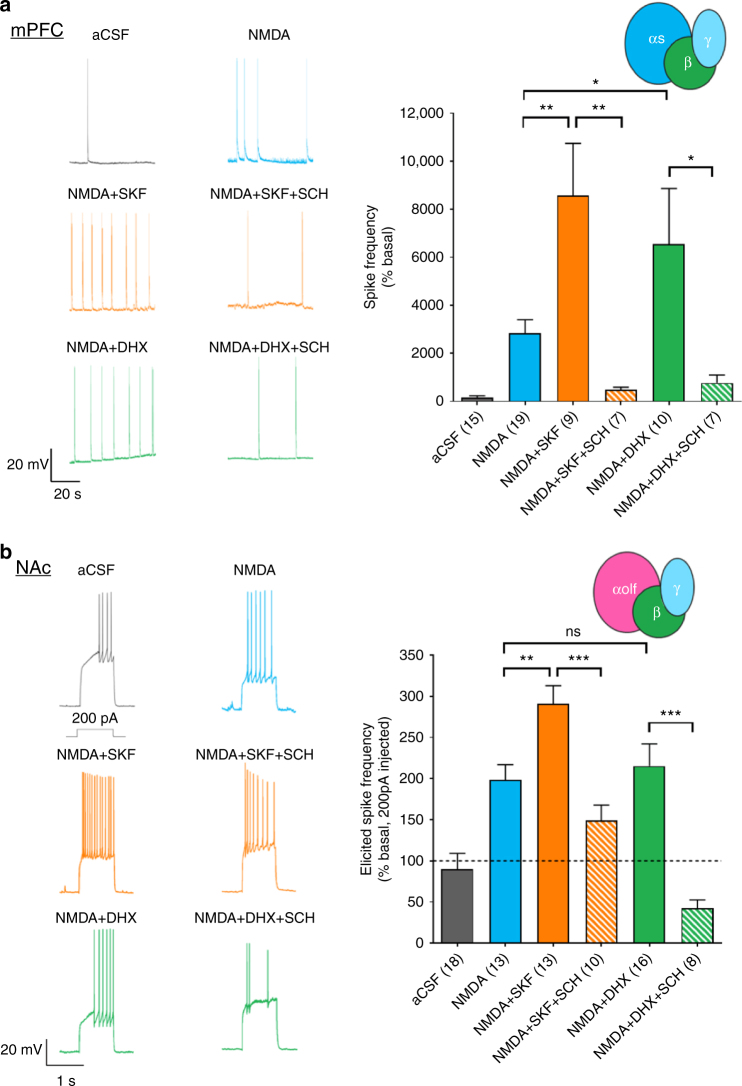
Current clamp recording of drug-induced firing events for D1R-expressing pyramidal neurons in mPFC (**a**) and medium spiny neurons in NAc (**b**). Bar graph shows compiled data for spike frequency over 10 min drug treatment (all 10 µM). For + SCH23390 condition, the antagonist is added in the aCSF and drug shown in the x-axis to ensure antagonist binding prior to the other drug’s effect. Example traces are shown on the left. Elicited spike frequency (200 pA injection via recording pipet) is shown for the NAc **b**. Number of cells recorded for each condition is shown in parenthesis. Values were statistically analyzed by one-way analysis of variance (ANOVA) repeated measure followed by Newman–Keuls post hoc test. *p*-values are as indicated: *p < 0.05, **p < 0.01, or ***p < 0.001; NS for not significant. The error bars represent S.E.M.

Increased neuronal excitability and cell firing mediated by D1R activation has been reported in the striatum^24–26^ as well as in the cortex^27–29^. D1R and NMDA receptors (NMDAR) have been reported to form molecular and functional interactions^30–32^ and D1R activation has been shown to facilitate NMDAR function via Gs/olf-AC-PKA activation^33^. Differences in Gs- and Golf-dependent effects of SKF81297 and DHX were therefore assessed by measuring NMDA-induced firing rates in D1R-expressing neurons using patch-clamp electrophysiology in slices from Drd1-tdTomato mice. First, firing rate was analyzed in D1R-expressing layer V pyramidal neurons in the Gs-enriched mPFC (Fig. 4a). The minimal basal spontaneous firing rate (0.010 ± 0.006 Hz) was dramatically increased by NMDA (10 µM; 0.298 ± 0.061 Hz; 2830% of basal), and this was further enhanced by co-application of SKF81297 (10 µM; 0.902 ± 0.229 Hz; 8570 % of basal; *p* < 0.01) or DHX (10 µM; 0.689 ± 0.244 Hz; 6540% of basal; *p* < 0.05). The enhancement of NMDA-induced firing by SKF81297 and DHX was blocked by the D1R antagonist SCH23390. Spontaneous firing, as well as NMDA-induced firing, was absent in NAc medium spiny neurons owing to their hyperpolarized resting membrane potentials. Therefore, in the Golf-enriched NAc, 200 pA was injected to elicit cell firing (Fig. 4b). NMDA (10 µM) robustly increased the elicited spike frequency (198% of control aCSF), and this was further enhanced by SKF81297 (291% of basal; *p* < 0.01 vs. NMDA). The effect of SKF81297 was inhibited by SCH23390. However, in contrast to the results obtained in the mPFC, DHX failed to further potentiate NMDA-induced increases in firing rate (215% of basal; *p* > 0.05 vs. NMDA). The D1R agonists by themselves, without NMDA application, did not enhance the firing rate in mPFC or NAc slices (Supplementary Fig. 7), indicating the need for concurrent NMDA-dependent cellular depolarization, although other ion channels may also be involved in the D1R agonist-mediated firing increase^34^. As previously reported^35^, SCH23390 partially inhibited the NMDA effect in both mPFC and NAc (Supplementary Fig. 7), indicating a possible direct interaction of SCH23390 with NMDAR as well as an effect from blocked basal DA tone. Together, these results suggest that the Gs-biased agonist DHX promotes unique effects on the activity of native neuronal cells that differentially express Gs and Golf.

### Weak psychomotor activating properties of DHX

**Fig. 5:**
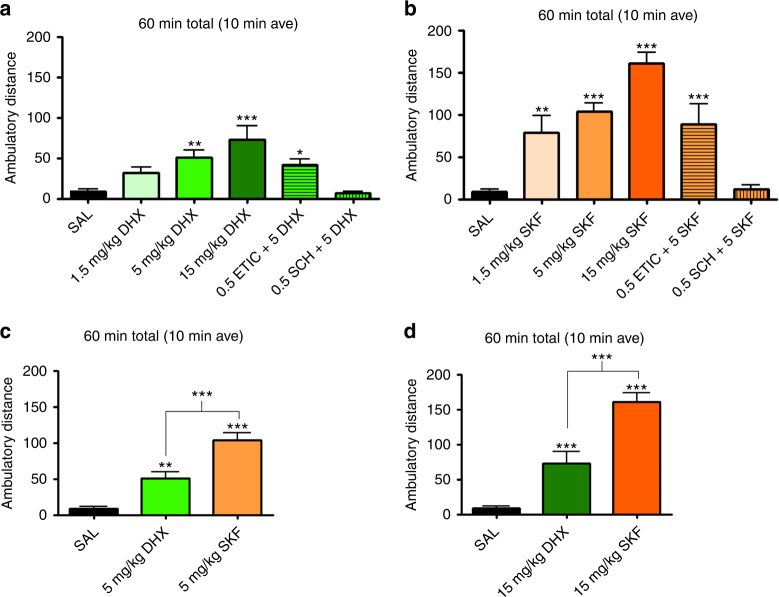
**a**, **b** Effect of D1R agonists on locomotion induced in reserpine-treated mice for DHX (**a**) and SKF81297 (**b**). Six bar group represents saline injection, 1.5 mg/kg, 5 mg/kg, 15 mg/kg, 5 mg/kg + 0.5 mg/kg eticlopride, 5 mg/kg + 0.5 mg/kg SCH23390 (left to right). **c**, **d** Comparison of the ambulatory distance between DHX and SKF81298 at 5 mg/kg (**c**) and 15 mg/kg (**d**). Values were statistically analyzed by one-way analysis of variance (ANOVA) followed by Newman-Keuls post hoc test. *p*-values are as indicated: *p < 0.05, **p < 0.01 or ***p < 0.001. The error bars represent S.E.M

Finally the ability of DHX to produce psychomotor activity by activating striatal D1R was explored in the catecholamine depleted long-term (twenty hours) reserpinized mouse model (Fig. 5)^36,37^. This model allows the in vivo determination of separate striatal post-synaptic activity of selective D1R and D2R agonists without the confounding influence of endogenous dopamine. Thus, either a selective D1R or a D2R agonist produces significant locomotor activation of the akinetic animal^36,37^. Both DHX and SKF81297 dose-dependently induced significant locomotor activation (Fig. 5a, b; see Supplementary Fig. 8B, C for time course), which was blocked by the D_1_-like receptor antagonist SCH23390 (0.5 mg/kg), but not by the D_2_-like antagonist eticlopride (0.5 mg/kg). The same dose of eticlopride, but not SCH23390, blocked quinpirole-induced locomotor activation (Supplementary Fig. 8A). These results demonstrated a selective involvement of D1R versus D2R in the locomotor activation and D1R-specific locomotor effects by DHX and SKF81297. Importantly, and consistent with its partial efficacy on the Golf-coupled striatal D1R, DHX showed a significantly lower locomotor-activating effect compared to the full agonist SKF81297 (Fig. 5c, d; 48.6% at 5 mg/kg or 45.8% at 15 mg/kg).

## Discussion

The development of D1R pharmacology for over 30 years has verified its potential therapeutic value in various neuropsychiatric disorders, including Parkinson’s disease, schizophrenia and substance use disorders. Intense research in GPCR pharmacology has yielded the concept of functional selectivity, giving rise to the possibility of selective targeting aside from receptor affinity, particularly the ability of ligands to preferentially activate either G-protein-mediated or β-arrestin-mediated signaling^5^. Initial insights into the structural basis for this functional selectivity are beginning to emerge^38^. The present study illustrates a unique paradigm for GPCR functional selectivity; namely, the differential ability of ligands to engage similar but distinct G-protein subtypes^13^. Following the same molecular principle as for the G-protein/β-arrestin functional selectivity, distinct conformations of the GPCR stabilized by a variety of ligands may also achieve G-protein subtype functional selectivity. Motivated by the unique reciprocal expression patterns of Gs and Golf in the cortex and striatum and the functional distinction in these brain areas, the current study was designed to look for D1R ligands that are functionally selective at Gs versus Golf.

DHX was developed in the late 1980’s^39^ and, based on results obtained with the cAMP assay in monkey and rat striatal tissue^40,41^ and behavioral effects in rats^42^, it was introduced as the first fully efficacious D1R agonist with potential antiparkinsonian effects. However, a clinical trial showed marginal therapeutic efficacy and secondary effects including hypotension and tachycardia^43^. The present study gives a new insight into the potential discrepancies previously observed with this drug and antiparkinsonian activity of DHX. We find that DHX indeed behaves as a full agonist at D1R, but only when coupled to Gs, not to the predominant striatal G-protein subtype Golf. Based on our molecular modeling study, we propose that the Gα/αN-D1R/IL2 interface plays a significant role in determining the reported biased agonism, as implicated by previous studies on differential ligand-induced conformations of the IL2 of monoaminergic receptors^44,45^. Our studies with the chimeric receptor suggest that D1R/IL2 may exist in distinct conformations such that DHX diminishes the already weak Gα/αN-D1R/IL2 interaction of D1R-Golf coupling, compared to DA. Although previous studies have reported a full efficacy profile reported for DHX, several caveats must be considered. First, the DHX efficacy has been reported in heterologous expression systems^46,47^, which rely mostly on Gs-mediated readout due to the fact that these cells do not express Golf. Second, the apparent full efficacy of DHX observed in experiments from Golf-rich striatal material^40,41^ could be related to the confounding effect of a concomitant Gs-dependent response from D1-like receptors localized in striatal neuronal populations other than medium spiny neurons, such as the large aspiny cholinergic interneurons. Indeed these interneurons are known to contain functional D1-like receptors of D5R subtype^48,49^. Finally, previous in vivo recordings from non-identified ventral pallidal neurons^50,51^ compared the effects of DHX with other D_1_-like receptor agonists. Single cell recording of D_1_-like receptor-expressing neurons in the striatum and cortex permitted us to better isolate the Golf- and Gs-dependent effects of D1-like receptor ligands. We could in fact confirm the significant respective predominance of Golf and Gs mRNA expression in these brain areas. We were then able to recapitulate by intracellular recordings the same differential pharmacological properties of DHX observed in vitro, namely striatal Golf-dependent efficacy differences between DHX and the full D1R agonist SKF81297.

Locomotor activation in reserpinized mice has been widely used to characterize striatal post-synaptic DA receptor pharmacology. With short-term reserpinization (about 4 h), D1R or D2R agonists produce no or little significant locomotor activation when administered alone, although a strong synergistic effect is observed upon co-administration of D1R and D2R agonists^36,37^. This situation parallels that in non-reserpinized mice, where endogenous dopamine synergizes with the effect of either D1R or D2R agonists. This explains the ability of either D1R or D2R antagonists to counteract the behavioral effects of both D1R or D2R agonists in non-reserpinized and short-term reserpinized mice^36,37^, as reported for DHX^42^. In contrast, with long-term reserpinization, synergistic effects of D_1_- and D_2_-like receptor agonists wane and administration of either agonist produces significant locomotion, which allows a more accurate pharmacological characterization of dopamine receptor ligands^36,37^. Long-term reserpinized mice were therefore used to establish the selective D_1_-like receptor partial agonistic profile of DHX. DHX produced a mild locomotor activation that was counteracted by the D_1_-like receptor antagonist SCH23390, but not by the D_2_-like receptor antagonist eticlopride, at the same dose that completely counteracted locomotor activation induced by the D2R agonist quinpirole. This is in spite of previous studies suggesting less potent but efficacious D_2_-like receptor agonistic properties of DHX^22,52^. A low potency toward D_2_-like receptors was also observed in the present study using BRET assays of D1R-, D2R-, and D3R-mediated G-protein activation and AC inhibition. In addition, in the G-protein activation BRET assays, DHX behaved as a partial D2R and D3R agonist as compared with the full agonist quinpirole (61.0% and 55.5% of quinpirole respectively), which may not be sufficient to trigger D_2_-like receptor-dependent locomotor activation in the reserpinized animal. The partial agonism of DHX in the reserpinized mice model thus confirms the key role of striatal post-synaptic Golf-dependent D1R in the mediation of locomotor activation induced by D1R agonists. In agreement with the electrophysiological experiments, we were able to recapitulate in vivo the lower efficacy of DHX, as compared to SKF81297, in an assay that depends on striatal D1R activation. Although differences in bioavailability between these two compounds cannot be ruled out, the lack of full efficacy with DHX is likely not due to poor brain availability, based on prior pharmacokinetic studies in rats^52^ as well as nonhuman primates^53^. In addition, various functional studies also exhibit a rapid onset of DHX activity in rodent brain^50,51,54^.

In summary, the current study provides the first example of a D1R agonist, DHX, that acts as a full agonist with Gs- and a partial agonist with Golf-dependent signaling. Consequently, DHX behaves as a low efficacy striatal D1R agonist but as a full D1R agonist in the cortex. This therefore provides a rationale for the use of DHX, or related compounds, in neuropsychiatric disorders with cognitive deficits, which could benefit from selective targeting of cortical D1R. Specifically, pharmacological enhancement D1R activity is being considered as a highly promising therapeutic mechanism for the amelioration of schizophrenia-spectrum cognitive deficits. Indeed, recent findings suggest that DHX can be potentially effective for the treatment of schizophrenia-spectrum working memory impairments^55^. The pharmacological characterization presented here, in combination with structure-activity relationship analysis, may lead to the development of compounds that can differentially affect D1R function in different brain regions.

## Methods

### DNA constructs

Human receptor constructs (D1R, D2SR, D3R, and muscarinic M1 receptor [M1R]) were modified N-terminally with in frame fusion of a signal peptide followed by a Flag or Myc epitope tag for enhanced cell surface expression^56^. D1R fused to *Renilla* Luciferase 8 (Rluc; provided by Dr. S. Gambhir, Stanford University, Stanford, CA) was described elsewhere^57^. The following non-fusion and fusion human G-protein constructs were used for cAMP accumulation assay and various bioluminescence resonance energy transfer (BRET) assays: Gαs short (Gαss), Gαolf, Gαss67-Venus, Gαss99-Venus, Gαss154-Venus, Gαolf69-Venus, Gαolf100-Venus, Gαolf155-Venus, Gαi191-Venus, Gαq150-Venus, Gαss67-Rluc, Gαolf69-Rluc, and GαoA91-Rluc (inserted positions noted). For Gγ2 and Gγ7 GFP10-fusion constructs, full-length GFP10 was fused at its N-terminus. Untagged βγ subunits Gβ2 and Gγ7 were also used for co-transfection. G-protein chaperone Ric8B^58,59^ (kind gift from Dr. Gregory Tall) was co-transfected with Gαss and Gαolf constructs. The cAMP sensor with YFP-Epac-Rluc (CAMYEL) was obtained from ATCC (no. MBA-277)^60^. G-protein receptor kinase 2 (GRK2) and mVenus-fused β-arrestin-2^61^ constructs were used for β-arrestin recruitment assay. Adenylate cyclase 5 (AC5, kind gift from Dr. Carmen Dessauer) was modified to generate AC5-NanoLuc (Promega) fusion construct. Both non-fusion and fusion AC5 constructs were used for the cAMP accumulation and BRET assays. All the constructs were confirmed by sequencing analysis.

### BRET assays

Variations of bioluminescence resonance energy transfer (BRET) assay were performed to detect receptor ligand-induced events. A constant amount of plasmid cDNA (15 µg) was transfected into human embryonic kidney cells 293 T (HEK-293T) using polyethylenimine (PEI; Sigma) in a 1:2 weight ratio in 10 cm plates. Cells were maintained in culture with Dulbecco’s modified Eagle’s medium (DMEM) supplemented with 10% fetal bovine serum (FBS, Atlanta), 2 mM L-glutamine (Gibco), and 1 % penicillin streptomycin (Gibco) and kept in an incubator at 37 °C and 5% CO_2_. The transfected amount and ratio among the receptor and heterotrimeric G proteins were tested for optimized dynamic range in drug-induced BRET. Experiments were performed approximately 48 h post-transfection. As reported previously^57^, cells were collected, washed, and resuspended in phosphate-buffered saline (PBS). Approximately 200,000 cells/well were distributed in 96-well plates, and 5 µM coelenterazine H (luciferase substrate, BRET1) or 5 µM coelenterazine 400a (luciferase substrate, BRET2) was added to each well. One minute after addition of coelenterazine, agonists were added to each well. Antagonists were added 15 min before the addition of agonist. Five different configurations of BRET were used: (i) Receptor-Gα engagement, (ii) Gα-γ protein activation, (iii) cAMP production, (iv) β-arrestin-2 recruitment, and (v) Gα-AC5 interaction. (i) Receptor-Gα engagement assay uses D1R-Rluc-Gαs-Venus or D1R-Rluc-Gαolf-Venus for a resonance energy transfer (RET) pair to study co-expressed D1R activity. (ii) Gα-γ protein activation assay uses Gαss-Rluc-γ7-GFP10, Gαolf-Rluc-γ7-GFP10, or Gαo-Rluc-γ2-GFP10 for a RET pair. Receptors and untagged Gβ2 constructs were co-transfected; (iii) cAMP production assay uses CAMYEL biosensor construct that contains Rluc and YFP allowing detection of intracellular cAMP change^60^ in conjunction with receptor co-expression. D1R-Gs activation was studied by agonist-induced cAMP increases. In order to study D2R-or D3R-Gi/o dependent cAMP inhibition activity, cells were pre-stimulated with 10 µM forskolin (Sigma) ten minutes prior to agonist treatment. (iv) β-arrestin-2 recruitment uses D1R-Rluc-β-arrestin-2-Venus for a RET pair. GRK2 was co-transfected to assist an enhanced phosphorylation required for the β-arrestin-2 recruitment. (v) Gα-AC5 interaction assay uses AC5-Nluc-Gαss-Venus or AC5Nluc-Gαolf-Venus as a RET pair to study D1R-induced events. The acceptor fluorescence was quantified. Venus was excited at 500 nm and measured at an emission wavelength of 530 nm. To confirm constant expression levels across experiments, GFP10 was excited at 405 nm measured at an wavelength emission of 515 nm. Both fluorophores were measured over 1 sec of recording, using a Mithras LB940 microplate reader (Berthold Technologies, Bad Wildbad, Germany). BRET1 signal from the same batch of cells was calculated as the ratio of the light emitted by Venus (530 nm) over that emitted by coelenterazine H (485 nm), and BRET2 signal from the same batch of cells was calculated as the ratio of the light emitted by GFP10 (515 nm) over that emitted by coelenterazine 400a (400 nm). BRET change was defined as BRET ratio for the corresponding drug minus BRET ratio in the absence of the drug. *E*_max_ values are expressed as the basal subtracted BRET change and in the dose-response graphs. Data and statistical analysis were performed with Prism 5 (GraphPad Software).

### cAMP accumulation assay in S49 cyc- cells

Mouse lymphoma S49 cyc- Tag cell line, a subclone of cyc- cells that stably expresses simian virus 40 (SV40) large T antigen (TAg), is used for electroporation and subsequent cAMP accumulation assay which is modified from previous study^20^. Cells are maintained in DMEM (Gibco) containing I0% FBS (Atlanta), 2 mM l-glutamine (Gibco), 1% penicillin streptomycin (Gibco) as well as 0.6 mg/ml of geneticin (GIBCO-BRL) to maintain expression of TAg and kept in an incubator at 37 °C and 5% CO_2_. Plasmids carrying D1R, Gαss or Gαolf, β2, γ7, Ric8B, and AC5 (45 µg total) are electroporated into the cells (10 million) by gene pulsar. cAMP assay is performed on cells 48 h post electroporation. Cells are resuspended in PBS subject to drug treatment—20 min agonist or the same with preceded 15 min antagonist incubation at room temperature. The incubation is stopped by centrifugation at 4 C, drug removal by aspiration, and cell lysis by 0.1 M HCl. Cell lysate samples are processed to measure cAMP levels by an enzyme-linked immunosorbent assay kit (Enzo Life Sciences, Farmingdale, NY) following manufacturer’s protocol. Protein concentration of the cells is determined by the quantitation assay (Pierce BCA protein assay, Thermo).

### Homology modeling and molecular dynamics simulation

Phylogenetically D1R is closer to the β2 adrenergic receptor (β2AR) than to any other member of the dopamine receptor subfamily^62^. Thus the models for human D1R-Gs and D1R-Golf complexes in active conformation were modeled based on the crystal structure of β2AR-Gs complex (PDB: 3SN6) bound to a full agonist BI-167107. The template (β2AR)—target (D1R) sequence alignment was extracted from G-protein-coupled receptor database (GPCRdb)^63^. The regions that do not have a template in the β2AR structure were not modeled. Five homology models for each of D1R-Gs and D1R-Golf complex were generated using MODELLER (version 9.17)^64^ and the models with the smallest objective function value were selected. To refine and validate the IL2 region of D1R, which is critical in the interaction with the Gs and Golf proteins (see text), we generated an ensemble of IL2 (residues 129–139 in D1R homology model) conformations (800 models) using the Random Coordinate Descent (RCD) algorithm implemented in RCD + webserver^65^. We compared the representative IL2 conformation from the lowest energy cluster of the ensemble with that of the selected homology models, and found they are similar. To further refine the models of the complexes, we used the side-chain prediction function^66^ of Prime program implemented in Schrödinger suite (release 2016–4, Schrödinger, LLC: New York, NY) to optimize the side-chain rotamers of the IL2 (residues 129–139) of the D1R and interacting residues on Gs and Golf (residues 28–40 and 30–42, respectively).

The optimized models were further investigated by molecular dynamics simulations. The simulation systems were built by embedding the D1R/G-protein complexes in the 1-palmitoyl- 2-oleoylphosphatidylcholine lipid bilayer solvent environment using Desmond (version 4.9; D. E. Shaw Research, New York, NY) with OPLS3 force field and TIP3P water model. The charge on the systems was neutralized by adding counter ions and 150 mM NaCl was added into the systems to attain physiological ionic strength. The system size is 519752 and 513427 atoms for D1R-Gs complex and D1R-Golf complex, respectively. Each system was first minimized and then equilibrated with restraints on the protein backbone-atoms, followed by an isothermal–isobaric simulation at 310 K with all atoms unrestrained, as described previously^67,68^. In the end, we collected 50 ns simulation time for each system.

### Animals

Male D1-tdtomato reporter BAC mice in C57BL/6 J background (Drd1-tdTomato line 6, Jackson Lab) were used for single cell reverse transcription PCR (RT–PCR) and slice electrophysiology experiments. Male wildtype C57BL/6 J mice were used for locomotor activity experiments. Animals were housed with food and water available ad libitum in temperature-controlled and humidity-controlled rooms and were maintained on a 12 h light/dark cycle. They were experimentally naive at the start of the study and were maintained under the approved protocol of the Institutional Care and Use Committee of the Intramural Research Program, National Institute on Drug Abuse.

### Single cell or tissue extraction RT–PCR

Quantitative real-time RT–PCR was performed and analyzed using LightCycler instrument 480 II (Roche). Coronal slices were prepared as described in slice electrophysiology. For D1R-expressing single cell analysis, Drd1-tdTomato mice were used. After identifying Drd1-tdTomato positive cells, cytoplasmic content was collected by micro pipet aspiration for single cell analyses. For tissue extracted analysis, wild type tissue samples were obtained in corresponding brain and processed for cell plasm. Collected cell plasm was immersed in buffer provided in a PicoPure RNA isolation kit (Invitrogen Life Technologies/Arcturus), and total RNA was isolated according to kit directions. Total RNA was converted to cDNA, then cDNA was amplified to antisense RNA (aRNA) by in vitro transcribe using the Message BOOSTER cDNA Synthesis Kit for qPCR (Epicentre Technologies, Madison, WI). The aRNA was purified using Qiagen RNeasy MinElute Cleanup kit and transcript to cDNA using SuperScript III Reverse Transcriptase (Invitrogen). PCR reactions were done in a total volume of 20 μL in PCR mix containing 10 μL 2X LightCycler probe master, 500 nM reference gene primer (GAPDH), 500 nM each of sense and antisense primer, 100 nM each of target gene probe and reference gene probe, and 5 μL of 100ng cDNA filled up to 20 μL with DEPC-treated H_2_O. Normalization of sample cDNA content was performed using the comparative threshold (ΔΔCT) cycle method, in which the number of target gene copies was normalized to an endogenous reference gene, GAPDH. CT is defined as the fractional cycle number at which the fluorescence generated by probe cleavage passes a fixed threshold baseline when amplification of the PCR product is first detected. The primers and probes were designed using Universal Probe Library Assay Design Center (Roche). Primer sequences and probes are as follows: 5′-gcagaaggacaagcaggtct-3′ (Gαs forward), 5′-gcttttgccagactctccag-3′ (Gαs reverse), 5′-atccgggatctgttcttgag-3′ (Gαolf forward), 5′-caggtgaagtgagggtagcag-3′ (Gαolf reverse), 5′-atggtgaaggtcggtgtga-3′ (GADPH forward), and 5′-aatctccactttgccactgc-3′ (GADPH reverse).

### Slice electrophysiology

Experiments were performed based on previous report with modifications^69^. Coronal slices (220 µm) were prepared from male adult Drd1-tdTomato mice using a vibrating tissue slicer (VT-1000S, Leica). Animals were anesthetized and perfused with modified artificial cerebral spinal fluid (m-aCSF) containing (in mM): 92 NMDG, 20 HEPES, 25 glucose, 30 NaHCO_3_, 1.2 NaH_2_PO_4_, 2.5 KCl, 5 sodium ascorbate, 3 sodium pyruvate, 2 thiourea, 10 MgSO_4_, 0.5 CaCl_2_, 300–310 mOsm, and pH 7.3–7.4. Slices were sectioned in cold m-aCSF and recovered at 32 °C in the same buffer saturated with 95% O_2_ and 5% CO_2_ (carbogen) for 10 min. Slices were then transferred to a holding chamber filled with carbogen saturated aCSF (holding aCSF) containing, in mM: 92 NaCl, 20 HEPES, 25 glucose, 30 NaHCO_3_, 1.2 NaH_2_PO_4_, 2.5 KCl, 5 sodium ascorbate, 3 sodium pyruvate, 2 thiourea, 1 MgSO_4_, 2 CaCl_2_, 300–310 mOsm, and pH 7.3–7.4. During recordings, slices were continuously perfused at 2 ml/min with carbogen-saturated aCSF containing (in mM): 124 NaCl, 2.5 KCl, 1.25 NaH_2_PO_4_, 1 MgCl_2_, 26 NaHCO_3_, 11 glucose, 2.4 CaCl_2_, 300–310 mOsm, and pH 7.3–7.4, supplemented with 200 µM sodium bisulfite, 100 µM picrotoxin and 10 µM eticlopride. The temperature of the recording chamber was maintained at 31–32 °C. Electrodes (3–5 MΩ) were backfilled with an internal solution containing (in mM): 120 mM K gluconate, 20 KCl, 0.05 EGTA, 10 HEPES, 1.5 MgCl_2_, 2.18 Na_2_ ATP, 0.38 Na GTP, 10.19 Na phosphocreatine, 280–285 mOsm, and pH 7.3–7.4. Cells were visualized on an upright microscope using infrared differential interference contrast video microscopy. Whole-cell current-clamp recordings were made using a MultiClamp 700B amplifier (2 kHz low-pass Bessel filter and 10 kHz digitization) with pClamp 10.5 software (Molecular Devices). Pyramidal neurons in the layer V prelimbic cortex and medium spiny neurons in the medial NAc shell were identified by morphology, membrane resistance, and hyperpolarized resting membrane potential. Series resistance (10–25 MΩ) was monitored with a 5 mV hyperpolarizing pulse (50 ms) given every 20 s, and only recordings that remained stable (monitored by series resistance) over the period of data collection were used. On breaking into neurons, the resting membrane potentials were between −70 and −90 mV. Current pulses (200 pA) were applied to medium spiny neurons using Clampex 10.5 and a MultiClamp 700B amplifier in current-clamp mode (Molecular Devices). All data are reported as mean ± SEM. Data was analyzed in Clampex and statistically analyzed with Prism 5 (GraphPad Software) by one-way ANOVA followed by Newman–Keuls post hoc test.

### Locomotor behavior assay

Experiments were based on a previous study^70^. Activity chambers with 42.0 × 42.0 cm open fields (Coulbourn Instruments, Allentown, PA) were used for the experiments with wild type C57BL/6 J mice. Reserpine (Sigma) was dissolved in a drop of glacial acetic acid, which was brought to volume with 5.5% glucose, then administered (5 mg/kg) subcutaneously 20 h prior to the start of the locomotor activity recording. All the other drugs (SKF81297, DHX, quinpirole, SCH23390, and eticlopride) were dissolved in sterile saline and administered intraperitoneally. Antagonists (SCH23390 or eticlopride) were administered 15 min prior to the locomotor activity recording and agonists (SKF81297, DHX, or quinpirole) were administered immediately before the animals were introduced in the open field for recording (at least *n* = 8 per each drug condition). All values (in cm of ambulation) registered per 10 min period were averaged for the first hour of recording. Different drug treated conditions were analyzed by one-way ANOVA followed by post hoc Tukey test.

### Data availability

All data that support the findings of this study are available from the corresponding author upon request.

## Electronic supplementary material

Supplementary Information
