# Supplementary material for: Gs- versus Golf-dependent functional selectivity mediated by the dopamine D1 receptor
Source: Nat Commun. 2018 Feb 5;9:486. doi: 10.1038/s41467-017-02606-w (PMC5799184; doi:10.1038/s41467-017-02606-w)
Supplement: Supplementary file 1 — Supplementary Information [file 41467_2017_2606_MOESM1_ESM.docx]

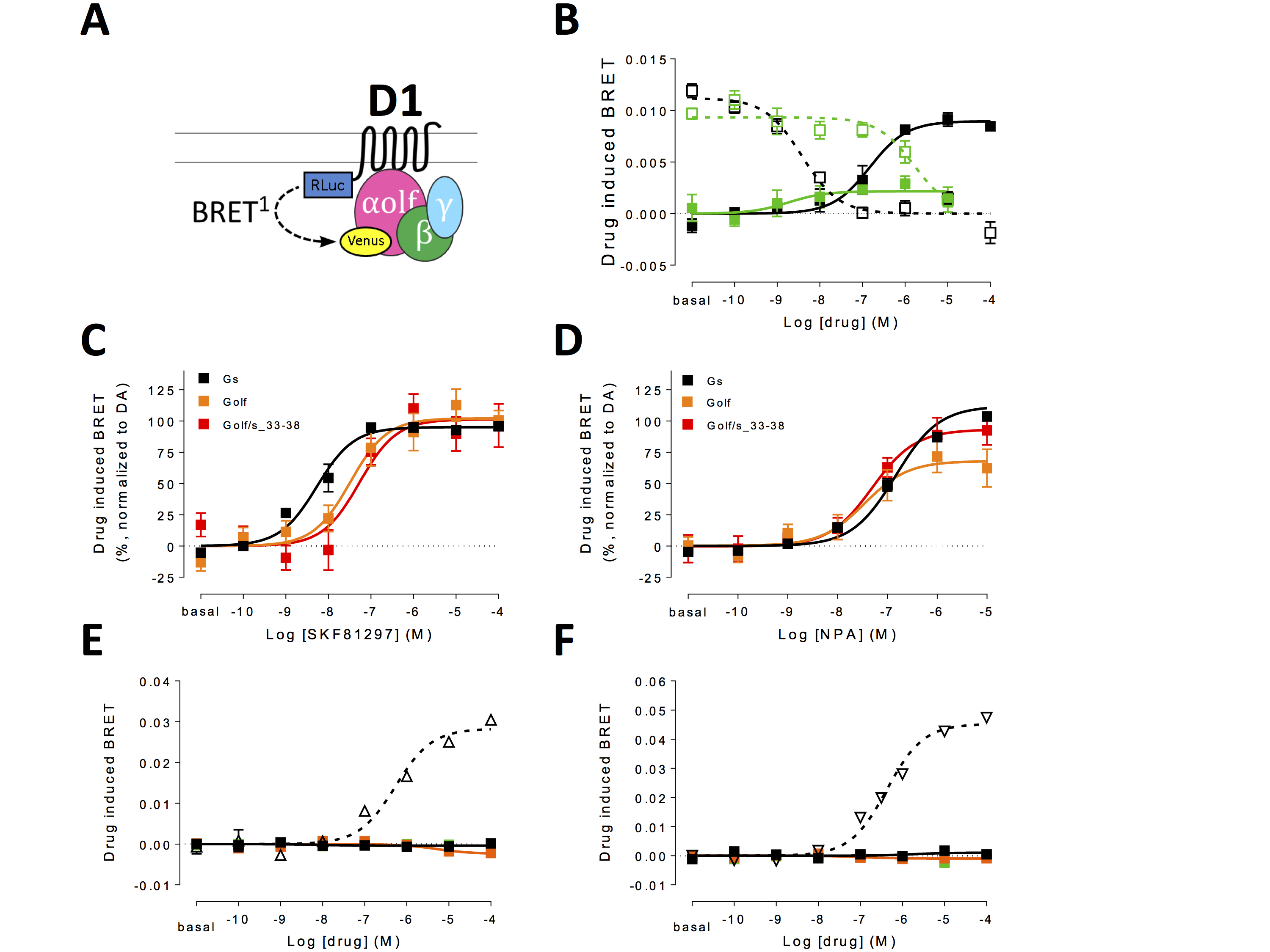


Supplementary Figure 1: **A**. Scheme for the engagement BRET between D1R-Rluc and Golf-Venus. **B**. Dose-response curves of drug induced BRET between D1R-Rluc and Golf-Venus (black, DA; green, DHX; black open, SCH23390 + 10^-6^ DA; green open, DHX + 10^-5^ DA). **C-D**. Dose-response curves of SKF81297 (**C**)- or NPA (**D**)- induced BRET between D1R-Rluc and Gs-Venus, Golf-Venus, or Golf/s_33-38-Venus (black, orange, and red respectively). BRET values are normalized to E_max_ values obtained by DA with corresponding Gα-Venus constructs. **E**. Dose-response curves of ligand induced BRET between D2R-Rluc and Gi1-Venus with DA (triangle) or D1R-Rluc and Gi1-Venus with DA (black square), SKF81297 (orange square) or DHX (green square). **F**. Dose-response curves of ligand induced BRET between M1R-Rluc and Gq-Venus with carbachol (triangle) or D1R-Rluc and Gi1-Venus with DA (black square), SKF81297 (orange square) or DHX (green square). The error bars represent S.E.M.


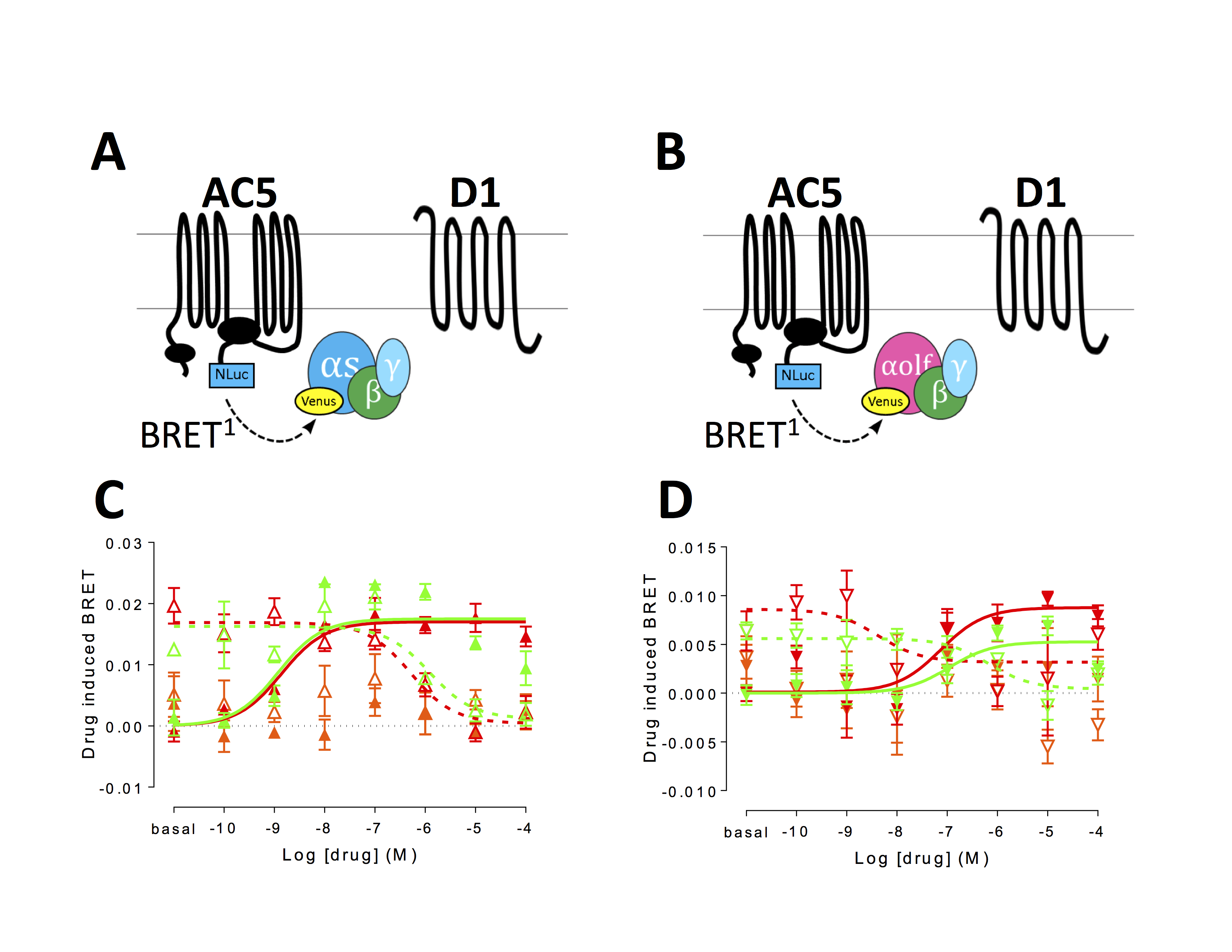


Supplementary Figure 2: **A**. Scheme for the drug-induced interaction BRET between AC5-Nluc and Gs-Venus. **B**. Scheme for the drug-induced interaction BRET between AC5-Nluc and Golf-Venus. **C**. Dose-response curves of drug induced BRET between AC5-Nluc and Gs-Venus for three different insertion positions (solid, DA; dashed, SCH23390 + 10^-6^ DA; orange, Gs67-Venus; red, Gs99-Venus; green, Gs154-Venus). **D**. Dose-response curves of drug induced BRET between AC5-Nluc and Golf-Venus for three different insertion positions (solid, DA; dashed, SCH23390 + 10^-6^ DA; orange, Golf69-Venus; red, Gs100-Venus; green, Golf155-Venus). The error bars represent S.E.M.


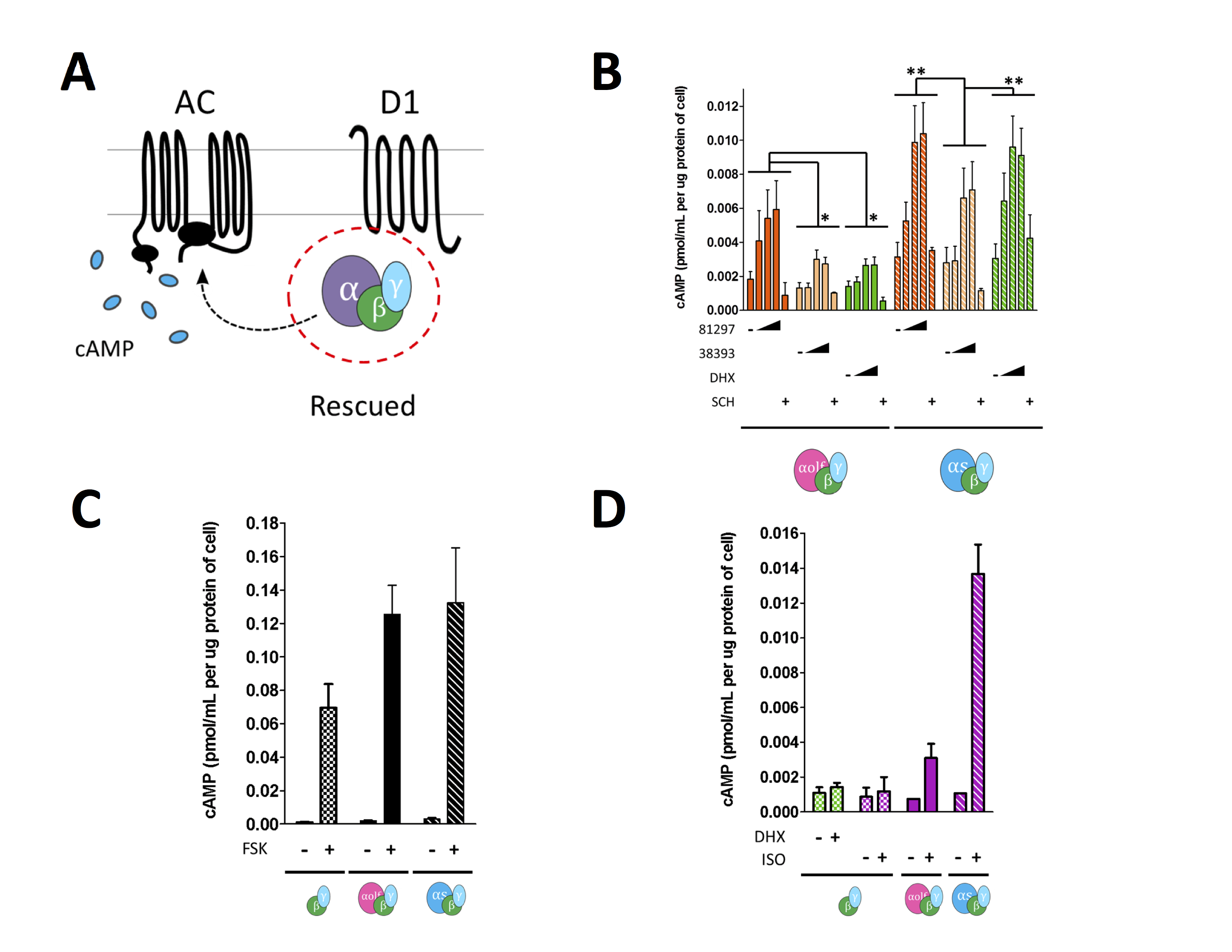


Supplementary Figure 3: **A**. Scheme for the cAMP accumulation assay in G protein rescued S49 cyc- cells. **B**. Dose-response bar graph of drug induced cAMP accumulation (solid = Golf rescue, hatch = Gs rescue, dark orange = SKF81297, light orange = SKF38393, green = DHX). Each five-bar group represents basal, 10^-9^, 10^-7^, 10^-5^, 10^-7^ + 10^-5^ SCH23390 (left to right). Values were statistically analyzed by one-way analysis of variance (ANOVA) repeated measure followed by Tukey post hoc test. p values are as indicated: * or ** : p<0.05 or 0.01. **C**. Forskolin response bar graph of cAMP accumulation (mosaic, no rescue; solid, Golf rescue; hatched, Gs rescue). Each two-bar group represents basal and 10^-5^ forskolin (left and right). **D**. Drug response bar graph of cAMP accumulation (mosaic, no rescue; solid, Golf rescue; hatched, Gs rescue). Each two-bar group represents basal and 10^-5^ of either DHX or isoproterenol (green or purple).


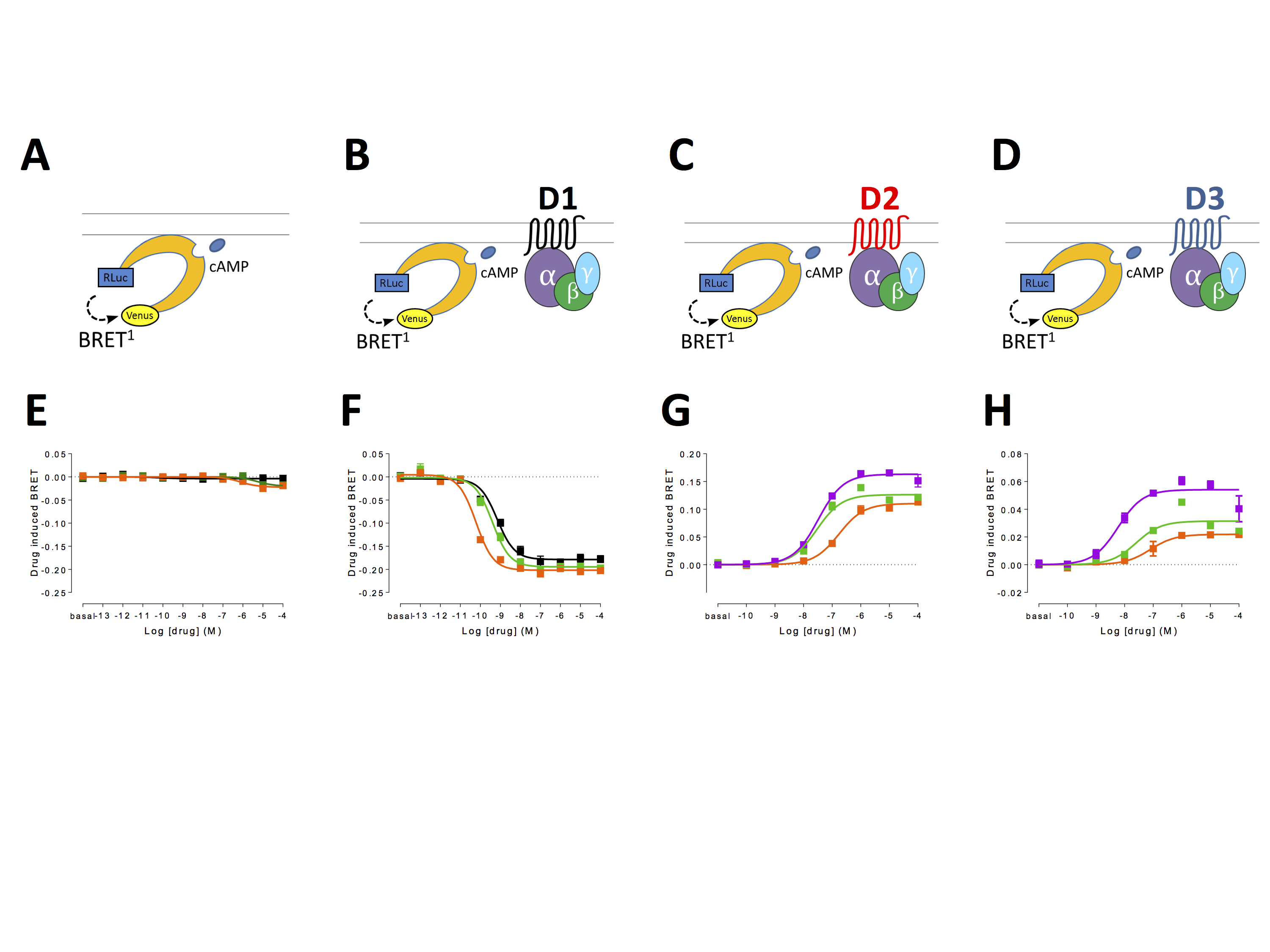
Supplementary Figure 4: **A-D**. Scheme for the drug-induced cAMP change detected by CAMYEL BRET sensor in non-D1R expressed cells (**A**), D1R expressing cells (**B**), D2R-expressing cells (**C**), and D3R-expressing cells (**D**). **E**. Dose-response curves of drug induced BRET decrease in non-D1R expressing cells (black, DA + 10^-6^ propranolol; green, DHX; dark orange, SKF81297). **F**. Dose-response curves of drug induced BRET decrease in D1R expressing cells (same color scheme as panel **E**). **G**. Dose-response curves of drug induced BRET increase in D2R-expressing cells (purple, 10^-5^ FSK + quinpirole; dark orange, 10^-5^ FSK + SKF81297; green, 10^-5^ FSK + DHX). **H**. Dose-response curves of drug induced BRET increase in D3R-expressed cells (same color scheme as panel **G**). The error bars represent S.E.M.


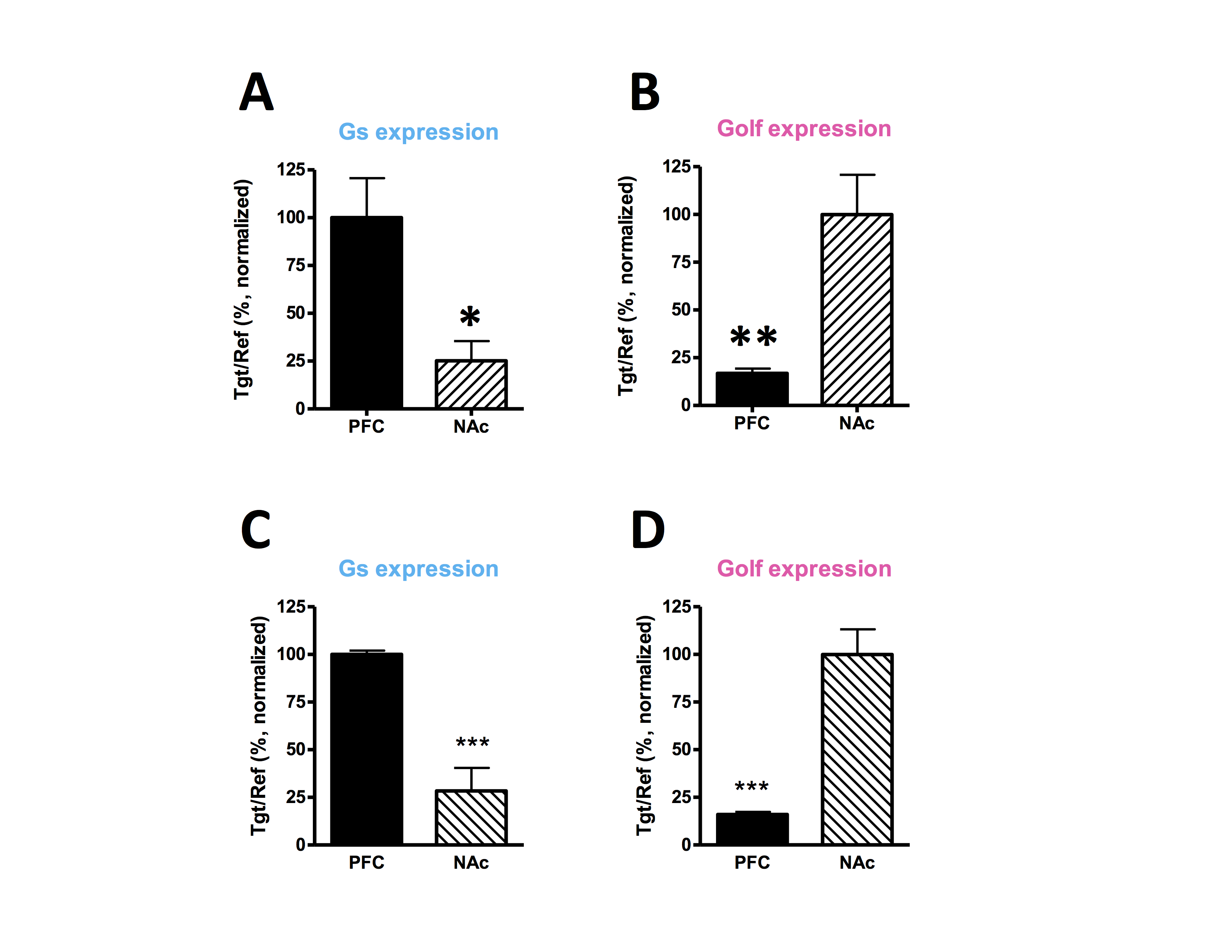


Supplementary Figure 5: **A-B**. Single cell mRNA levels of D1-tdTomato reporter mouse for Gs (**A**) and Golf (**B**) expression in pyramidal cells of mPFC (solid) and medium spiny cells of NAc (hatch). Results are average of four cells and normalized to mPFC (**A**) and NAc (**B**). **C-D**. Tissue-extracted mRNA levels of wild type mouse for Gs (**C**) and Golf (**D**) expression in mPFC (solid) and NAc (hatch). Results are average of nine samples and normalized to mPFC (**C**) and NAc (**D**). Values were statistically analyzed by t test. p values are as indicated: *,** or ***: p<0.05, 0.01 or 0.001. The error bars represent S.E.M.


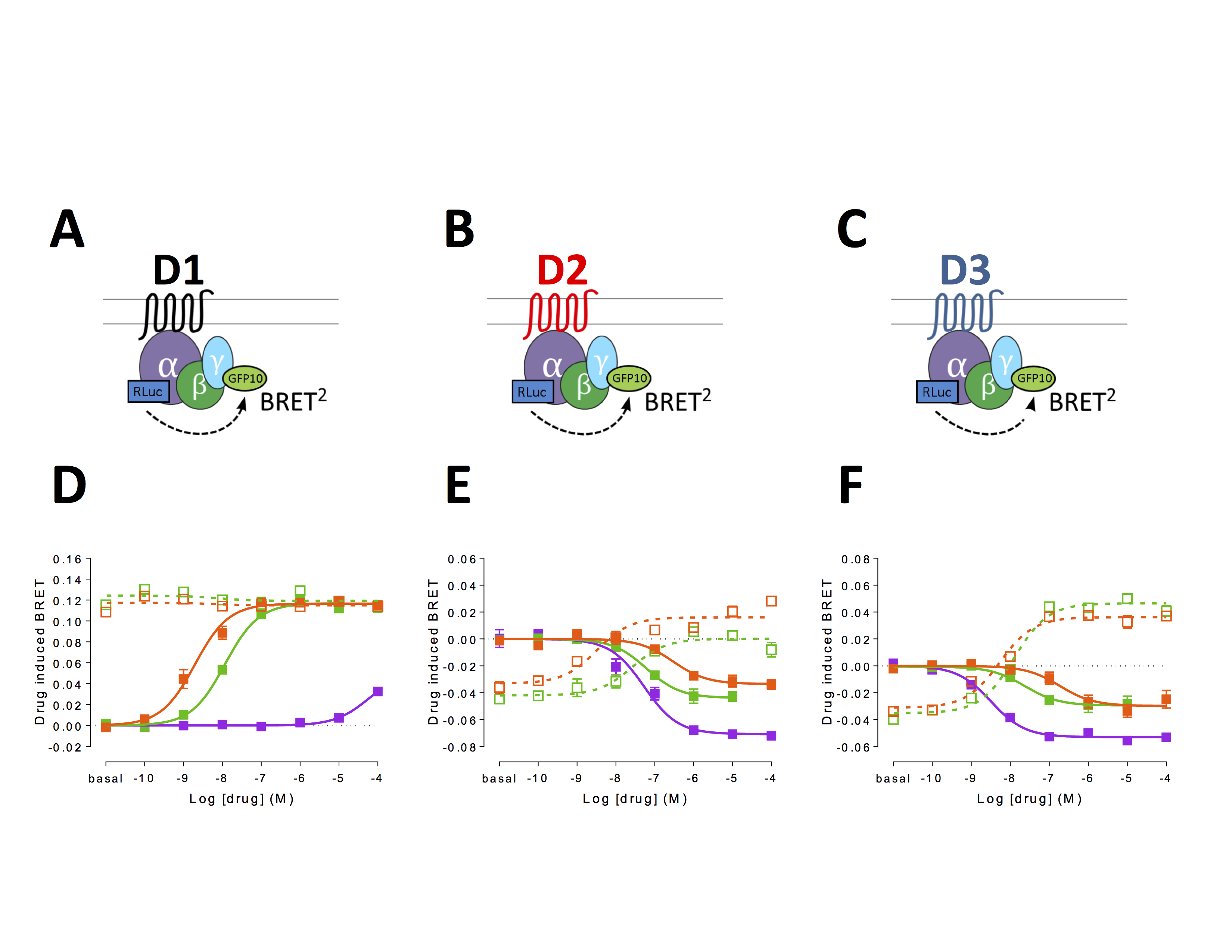


Supplementary Figure 6: **A-C**. Schemes for the G protein activation BRET. **D**. Dose-response curves of D1R mediated Gs activation BRET (purple = quinpirole, green = DHX, dark orange = SKF81297, green open = eticlopride + 10^-6^ DHX, dark orange open = eticlopride + 10^-6^ SKF81297). **E**. Dose-response curves of D2R mediated Go activation BRET (same color scheme as panel D). **F**. Dose-response curves of D3R mediated Go activation BRET (same color scheme as panel D). The error bars represent S.E.M.


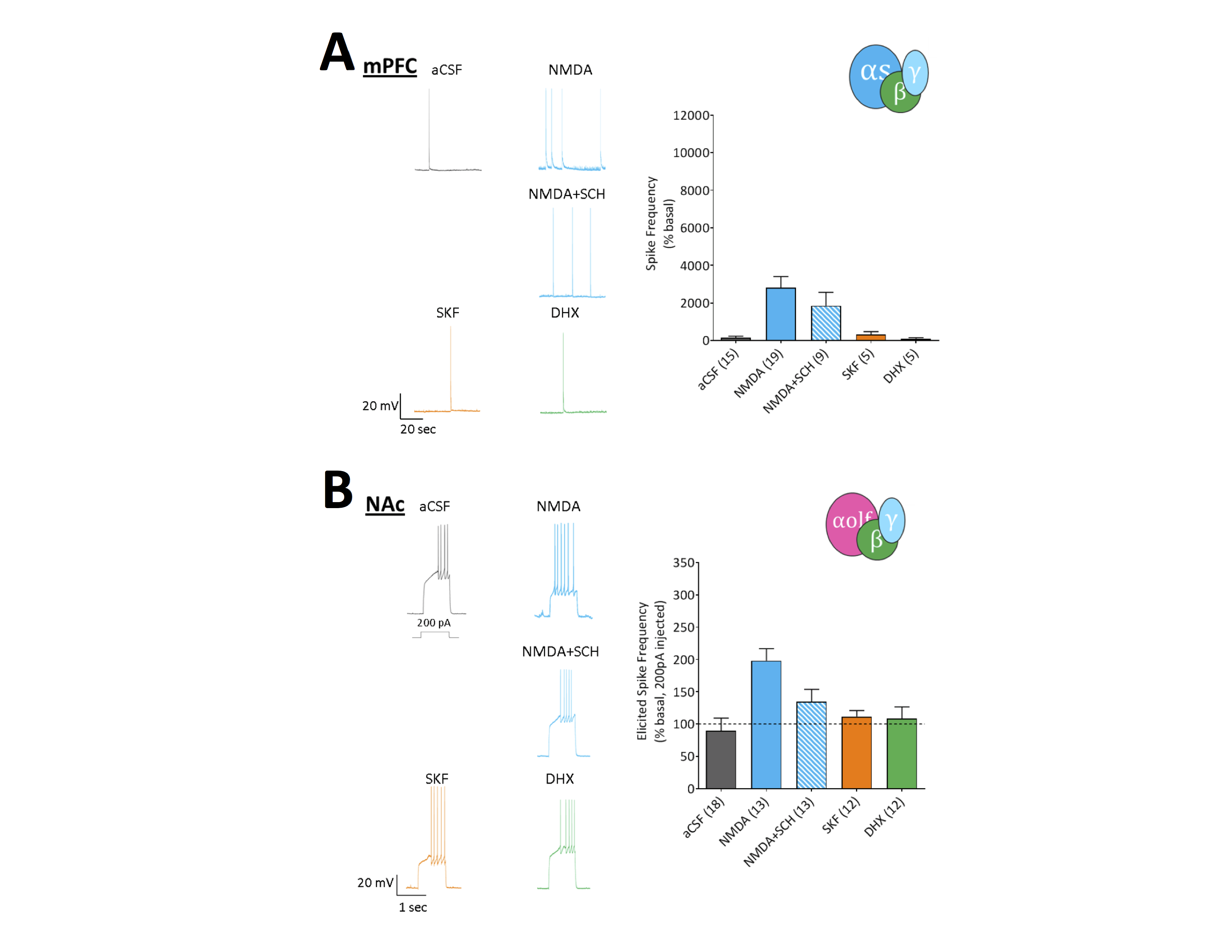


Supplementary Figure 7: Current clamp recording of drug induced firing events for D1R-expressing pyramidal neurons in mPFC (**A**) and medium spiny neurons in NAc (**B**). Bar graph shows compiled data for spike frequency over 10 min drug treatment (all 10 µM). For +SCH23390 condition, the antagonist is added in the aCSF and drug shown in the x-axis to ensure antagonist binding prior to the other drug’s effect. Example traces are shown on the left. Elicited spike frequency (200 pA injection via recording pipet) is shown for the NAc (**B**). Number of cells recorded for each condition is shown in parenthesis. The error bars represent S.E.M.


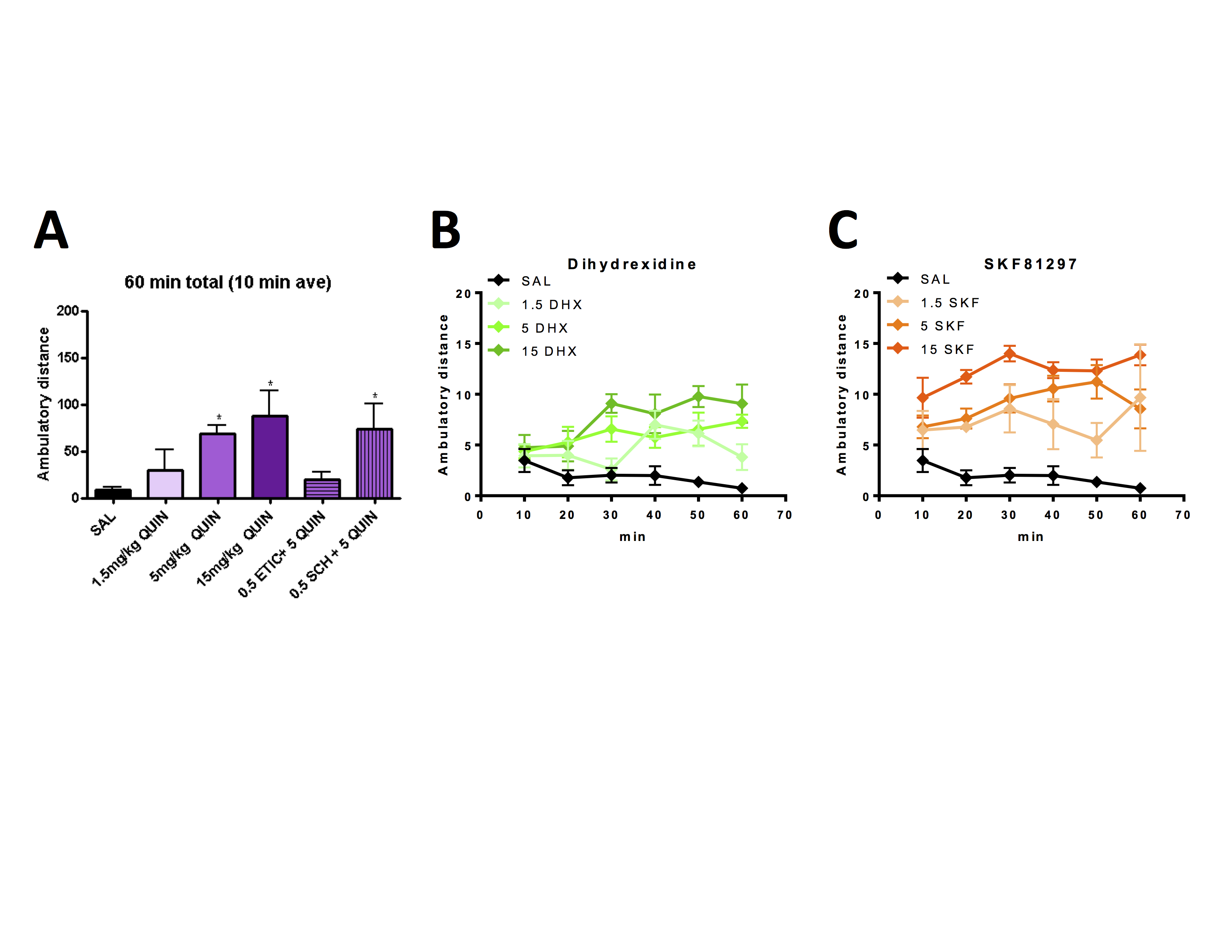
Supplementary Figure 8: **A**. Effect of D2-like receptor agonist quinpirole on locomotion induced in reserpine-treated mice. Six bar group represents saline injection, 1.5 mg/kg, 5 mg/kg, 15 mg/kg, 5 mg/kg + 0.5 mg/kg eticlopride, 5 mg/kg + 0.5 mg/kg SCH23390 (left to right). Values were statistically analyzed by one-way analysis of variance (ANOVA) followed by Newman-Keuls post hoc test. p values are as indicated: *: p<0.05. **B-C**. Time course representation of locomotor effects by DHX (**B**) or SKF81297 (**C**). The error bars represent S.E.M.


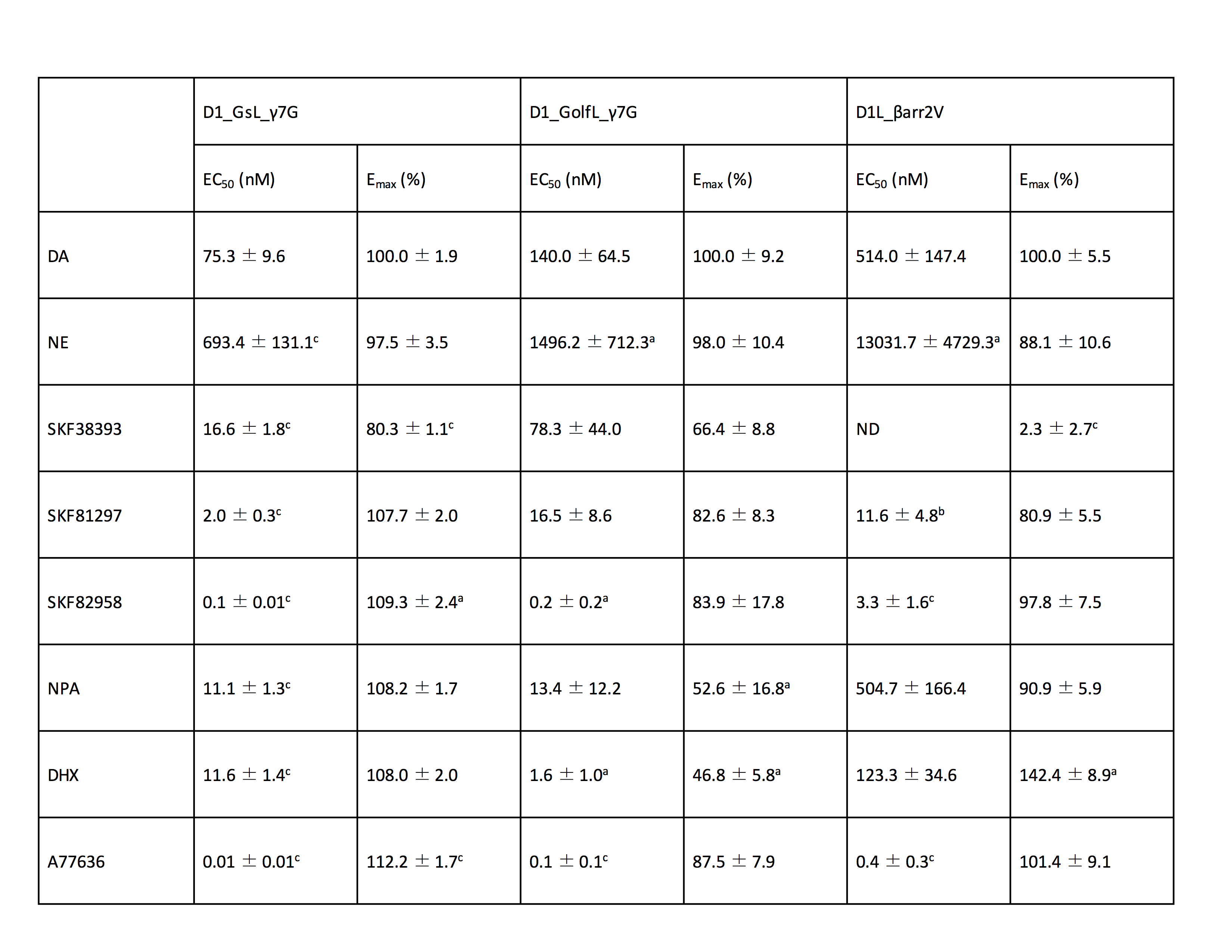


Supplementary Table 1: Pharmacological comparison of Gs and Golf activation by D1R and β arrestin-2 recruitment to D1R. Data were fit by non-linear regression to a sigmoidal dose-response relationship against the agonist concentration. EC_50_ and E_max_ values are means ± S.E.M. of more than 5 experiments performed in triplicate. E_max_ values are expressed in % normalized to dopamine results. ^a^P < 0.05, ^b^P < 0.01, ^c^P < 0.001 compared with DA (one-way analysis of variance, followed by post hoc Tukey test).


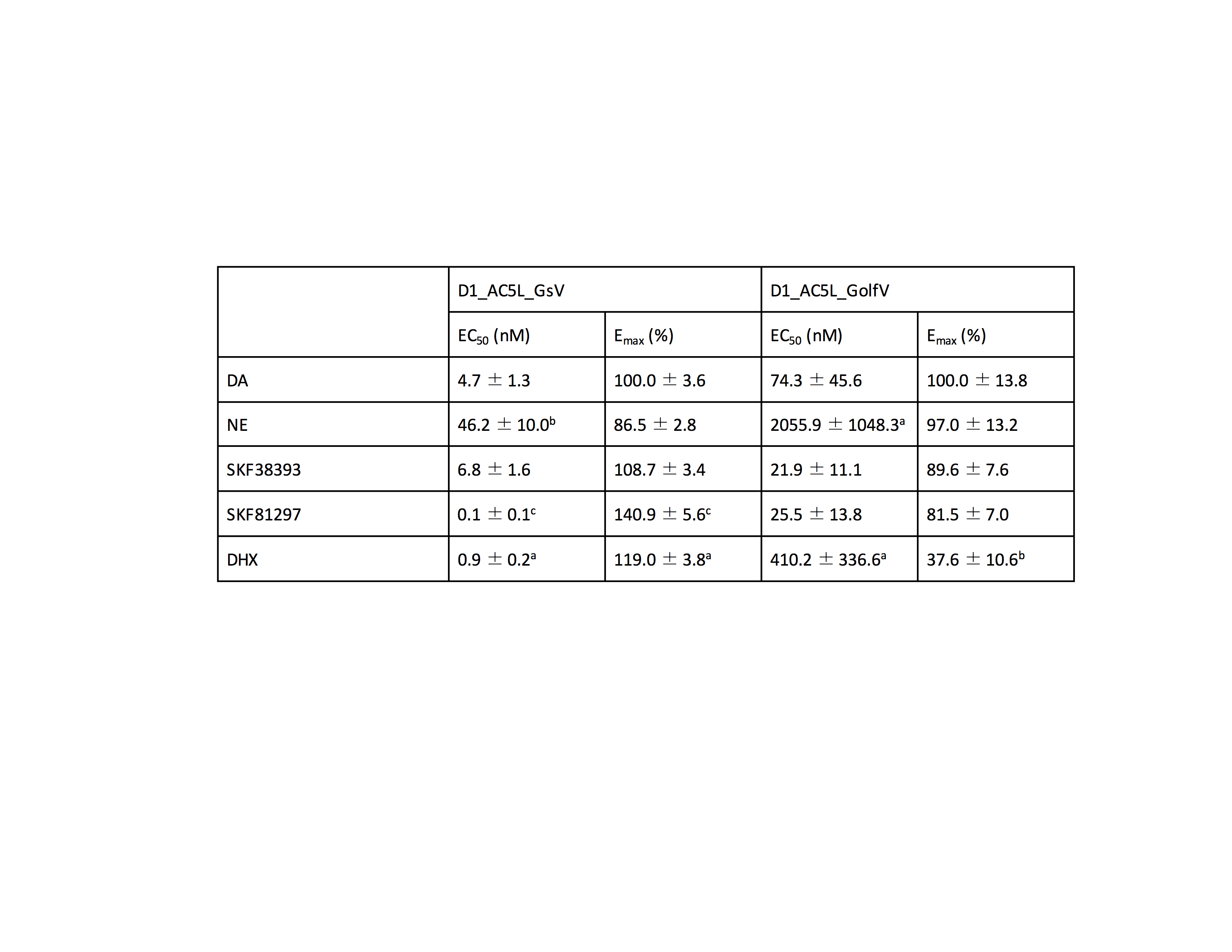


Supplementary Table 2: Pharmacological comparison of Gs and Golf engagement to AC5 by D1R activation. Data were fit by non-linear regression to a sigmoidal dose-response relationship against the agonist concentration. EC_50_ and E_max_ values are means ± S.E.M. of more than 5 experiments performed in triplicate. E_max_ values are expressed in % normalized to dopamine results. ^a^P < 0.05, ^b^P < 0.01, ^c^P < 0.001 compared with DA (one-way analysis of variance, followed by post hoc Tukey test).
